# Strong Saharan Dust Deposition Events Alter Microbial Diversity and Composition in Sediments of High-Mountain Lakes of Sierra Nevada (Spain)

**DOI:** 10.1007/s00248-024-02416-w

**Published:** 2024-07-27

**Authors:** Antonio Castellano-Hinojosa, Germán Tortosa, Alejandra Fernández-Zambrano, David Correa-Galeote, Eulogio J. Bedmar, Juan M. Medina-Sánchez

**Affiliations:** 1 Instituto Universitario de Investigación del Agua, Universidad de Granada, Granada, Spain; 2 Departamento de Microbiología, Facultad de Farmacia, Universidad de Granada, Granada, Spain; 3 Departamento de Microbiología del Suelo y Sistemas Simbióticos, Estación Experimental del Zaidín, Consejo Superior de Investigaciones Científicas, Granada, Spain; 4 Departamento de Ecología, Facultad de Ciencias, Universidad de Granada, Granada, Spain

**Keywords:** Freshwater ecosystems, Atmospheric deposition, Sediments, Nutrients, Microbial diversity

## Abstract

Mediterranean high-mountain lakes are being increasingly affected by strong Saharan dust deposition events. However, the ecological impacts of these severe atmospheric episodes remain largely unknown. We examined the effects of a strong Saharan dust intrusion to the Iberian Peninsula in 2022 on the physicochemical parameters and prokaryotic communities in sediments of nine high-mountain lakes of Sierra Nevada (Spain) located above 2800 m.a.s.l and in different orientations (north vs. south). A previous year (2021), with lower Saharan dust deposition with respect to 2022, was used for interannual comparisons. The strong dust deposition to the high-mountain lakes resulted in a significant increase in sediment nutrient availability which was linked to changes in the composition of prokaryotic communities. Decreases in alpha diversity and changes in beta diversity of prokaryotic communities were mainly observed in lakes located in the south compared to the north orientation likely because the former was more affected by the atmospheric dust deposition episode. Dust intrusion to the high-mountain lakes resulted in significant changes in the relative abundance of specific genera involved in important nutrient cycling processes such as phosphate solubilization, nitrogen fixation, nitrification, and denitrification. Saharan dust deposition also increased predicted microbial functionality in all lakes. Our findings show that severe atmospheric dust inputs to remote high-mountain lakes of Sierra Nevada can have significant biogeochemical and biodiversity consequences through changes in nutrient availability and prokaryotic communities in sediments of these freshwater ecosystems. This information contributes to understanding how Mediterranean high-mountain lakes of Sierra Nevada face strong intrusions of Saharan dust and their ecological consequences.

**Supplementary Information:**

The online version contains supplementary material available at 10.1007/s00248-024-02416-w.

## Introduction

Atmospheric dust deposition represents a significant source of inorganic (mainly phosphorus) and organic nutrients to high-mountain freshwater ecosystems and has received increasing attention as an important agent of climate change [1, 2]. These aerosols are suspended particles and can vary in their composition (e.g., biological or mineral), size (0.001–100 µm), and shape [3, 4]. Aeolian dust particles can also serve as a means for transporting microorganisms over long distances which may influence local communities in recipient high-mountain freshwater ecosystems [2, 5]. The Sahara-Sahel region in Northern Africa is a major global source of dust in the Northern Hemisphere [6]. For example, research suggests that over half of the global mineral dust aerosols originate from this specific area, with an estimated annual transport of 80–120 Tg northward across the Mediterranean Sea to Europe [7]. Mediterranean high-mountain areas appear to be increasingly affected by strong Saharan dust deposition events [8].

Sierra Nevada extends from west to east over a distance of 80 km, includes the highest peaks of the Iberian Peninsula standing at elevations above 3300–3400 m.a.s.l. [9], and constitutes a hot spot for biodiversity in Southern Europe [10]. Notably, Sierra Nevada’s high-mountain lakes are located above the tree line, at altitudes near 3000 m.a.s.l., and are considered true sentinels of global change owing to their elevation, geographical isolation, remoteness, limited drainage areas, intense ultraviolet radiation exposure, and the wide variety of water bodies found within a small mountainous area [11]. Sierra Nevada, because of its elevation, acts as a major obstacle that intercepts the Saharan dust transported northward from North Africa in the upper atmosphere between 1500 and 4000 m.a.s.l., specifically above the planetary boundary layer (with a mean annual height of 1700 ± 500 m.a.s.l. [2, 12]). Atmospheric deposition with Saharan dust is reported to introduce organic matter and nutrients such as nitrogen (N), phosphorus (P), calcium (Ca), and iron (Fe) to Sierra Nevada high-mountain lakes which subsequently impact lake primary and bacterial productivity [2, 13]. In addition, it has been reported that bacteria from the Sahara Desert are deposited to high-mountain lakes in Sierra Nevada through dust depositions at a rate from 3 × 10^6^ to > 80 × 10^6^ cells m^−2^ per day, particularly during rainy events [14]. Saharan dust was shown to have a significant positive effect on bacterial abundance but not on the richness, diversity, or composition of indigenous bacterial communities in ex situ microcosm experiments using water from a reservoir at a low altitude in Sierra Nevada [15]. More recently, in situ experiments in a high-mountain lake of Sierra Nevada (La Caldera Lake) showed significant increases in bacterial abundance after Saharan dust addition (Vila et al. submitted). Yet more in situ studies on the effect of Saharan dust deposition events on microbial communities in sediments of high-mountain lakes are needed to better understand the relevance of these events to changes in nutrient cycling and environmental microbiomes in these remote areas.

A severe Saharan dust intrusion to the Iberian Peninsula occurred in March 2022 which was identified of high intensity and large geographical extent [8, 16, 17]. This dust event extended beyond the Iberian Peninsula, crossing the Pyrenees and spreading into France, Switzerland, and the UK [16, 17]. This strong Saharan dust episode was the highest registered in Sierra Nevada over the past 40 years (Supplementary Fig. S1) [8, 16, 17]. In fact, the historical MERRA-2 record for the area Granada-Sierra Nevada showed surface concentrations of dust particles greater than 50 µg/m^3^ and 100 µg/m^3^ during more than 77 and 35 days, respectively, in 2022 [8, 17]. This year had the highest number of days registering these extreme dust concentrations, harmful to human health, in the last 40 years in Sierra Nevada [8, 18]. The massive amount of Saharan dust particles deposited in 2022 to Sierra Nevada’s high-mountain lakes provides a unique opportunity to determine the relevance of these events compared to previous years with lower atmospheric dust deposition (Supplementary Fig. S1) [17]. For example, the study of changes in nutrient availability and microbial communities in high-mountain freshwater ecosystems of Sierra Nevada can help better understand the biogeochemical consequences of severe atmospheric inputs which can contribute to facing future environmental changes.

Prokaryotic communities are essential components in the oligotrophic high-mountain lakes of Sierra Nevada [11]. For example, they play a key role in carbon cycling in heterotrophic microbial food webs [9, 19] and in the removal of reactive N species (Nr) mainly via denitrification [20, 21], among other processes. Previous studies have found that Saharan dust intrusion events can impact the bacterial community composition of the bacterioneuston (bacteria inhabiting the air–water interface) in high-mountain lakes of the Pyrenees [22–24] and Austrian and Italian Alps [25, 26]. Saharan dust intrusions can significantly impact bacterial productivity in Mediterranean high-altitude lakes by delivering inorganic nutrients and organic carbon through deposition [15, 27–29]. However, less is known about the potential linkage between changes in nutrient availability from lake sediments and variations in the diversity, composition, and functionality of microbial communities after strong Saharan dust deposition events in high-mountain lakes. This information can help identify responsive taxa to atmospheric inputs and key drivers controlling variations in biogeochemical cycles in these remote high-mountain lakes that act as sensors of global change.

The goal of this study was to examine the effect of a strong Saharan dust deposition event in 2022 on the physicochemical parameters of the sediment and the diversity, composition, and predicted functionality of prokaryotic communities in sediments of nine high-mountain lakes of Sierra Nevada (Spain) located above 2800 m.a.s.l and in different orientations (north vs. south). A previous year (2021), with lower Saharan dust deposition respect to 2022, was used for interannual comparisons (Supplementary Fig. S1). Prokaryotic taxa responsive to the atmospheric deposition event were identified, and their relative abundances were linked to variations in sediment physicochemical parameters and lake’s orientation. We hypothesized that a strong Saharan dust deposition event in the high-mountain lake of Sierra Nevada will (i) promote increases in nutrient availability, (ii) alter the diversity and composition of prokaryotic communities due to the enhanced nutrient levels, and (iii) promote increases in the relative abundance of specific taxa and predicted functions related to changes in nutrient cycling compared to a year with lower atmospheric dust deposition. We also hypothesized that the extent of these effects will be dependent on the orientation of the mountain slopes on which lakes are located. The lakes located in the south orientation are expected to receive a greater concentration of nutrients by northward atmospheric dust intrusions compared to lakes located in the north orientation.

## Materials and Methods

### Study Site and Saharan Dust Deposition Events in High-Mountain Lakes

Nine high-mountain lakes located in the Mediterranean mountain of Sierra Nevada (Granada, Spain) were selected for this study (Supplementary Fig. S2). The selected lakes and their orientation were La Caldera (CAL; south), Río Seco (RS1; south), Poqueira 1 (PQ1; south), Poqueira 2 (PQ2; south), Aguas Verdes (LAV; south), Virgen Media (LVM; north), Virgen Grande (LVG; north), Lagunillo Alto (LTA; north), and Lagunillo Bajo (LTB; north). Additional details about the location and altitude of these lakes are presented in Table 1. The lakes are situated on a siliceous substrate, such as shales and quartzites, which is part of the Mulhacén mantle of the Nevado-Filábride complex of the Betic ranges. Additional geological information about the lakes can be found in Santamaría-López et al. [30]. In Sierra Nevada, rainfall increases from east to west, and the average annual rainfall ranges between 600 and 900 mm at 2000 m. Moreover, about 80% of this precipitation typically falls between the months of October and April [31]. Above 2500 m.a.s.l., the average precipitation is 889 mm per year, 59% of which occurs as snow [32]. The interannual variability of surface dust concentration between 1980 and 2022 in Sierra Nevada is presented in Supplementary Fig. S1, and additional details are provided in Smart Ecomountains [17]. An increasing trend in surface dust concentration has been observed in the Sierra Nevada mountain from 1980 to 2022 (Supplementary Fig. S1) [17]. A collection of pictures of all high-mountain lakes of Sierra Nevada, classified by valleys and dates covering at least the last two decades, can be found on the website https://lagunasdesierranevada.es/lagunas/. An image of La Caldera Lake (one of the most studied lakes of Sierra Nevada), showing a strong color change from “normal” clear and blue (before 2022) to muddy and brown after the strong Saharan deposition in 2022, can be found in Biddanda et al. [33].

**Table 1:** Details about the location, elevation, orientation, and acronym used for the nine lakes selected for this study

Lake*	Acronym	Location (GPS coordinates)	Altitude (m.a.s.l.)	Orientation
La Caldera	CAL	X: 37.054566–Y: − 3.329206	3050	South
Rio Seco	RS1	X: 37.052113–Y: − 3.345583	2987	South
Poqueira 1	PQ1	X: 37.050663–Y: − 3.323312	2985	South
Poqueira 2	PQ2	X: 37.048073–Y: − 3.323628	2879	South
Aguas Verdes	LAV	X: 37.048674–Y: − 3.368381	3085	South
Virgen Medio	LVM	X: 37.051967–Y: − 3.379714	2939	North
Virgen Grande	LVG	X: 37.050881–Y: − 3.379955	2973	North
Lagunillo Alto	LTA	X: 37.051912–Y: − 3.378810	2956	North
Lagunillo Bajo	LTB	X: 37.052645–Y: − 3.378861	2930	North

^*^The official names of the lakes according to https://lagunasdesierranevada.es are CAL, Laguna de la Caldera; RS1, Laguna de Rio Seco; PQ1, Lagunillo del Río Mulhacén (official name under evaluation); PQ2, Lagunillo del Majano; LAV, Laguna de Aguas Verdes; LVM, Lagunillo de la Virgen que drena aguas abajo de las Yeguas; LVG, Lagunillo grande de la Virgen; LTA, Lagunillo de la Virgen tributario a la laguna de las Yeguas (alto); LTB, Lagunillo de la Virgen tributario a la laguna de las Yeguas (bajo)

### Sampling and Physicochemical Analyses in Sediment Samples

Four sampling locations were defined in each lake. Sampling was performed during the ice-free period between September and October in 2021 and 2022. Sediment samples were taken directly with 50-mL sterile falcon tubes from the sediment surface (0 to 5 cm depth). Samples were kept refrigerated (~ 4 °C) and transported to the laboratory. Each sediment sample was divided into two subsamples, one for sediment physicochemical analyses (kept at 4 °C) and the other for microbial analyses (kept at − 80 °C). The concentration of sodium (Na^+^), ammonium (NH_4_^+^), potassium (K^+^), magnesium (Mg^2+^), calcium (Ca^2+^), nitrite (NO_2_^−^), nitrate (NO_3_^−^), phosphate (PO₄^3−^), and sulfate (SO₄^2−^) was determined by ionic chromatography (Metrohm IC Compact 761). Sediment samples were extracted with distilled water (1:10, w/v) or 1 N KCl (1:10, w/v, only for NH_4_^+^, NO_2_^−^, and NO_3_^−^ analyses), shaken for 2 h at 170 rpm, centrifuged at 4000 rpm for 10 min, and filtered through 0.22 µm before analysis. Blanks were included during ion concentration measurements and showed no contamination. Standard curves were run for each ion and fit well, with *R*^2^ values ranging from 0.992 to 0.998. All detection values in the samples fell within the concentration range of the standard curves. Total carbon (TC), total organic carbon (TOC), and total nitrogen (TN) were measured using a LECO TruSpec CN elemental analyzer. pH and electrical conductivity (EC) were determined after water extraction (1:5, w/v) for 2 h.

### DNA Extraction

Total DNA was extracted from 500 mg of sediment using the FastDNA SPINK Kit for Soil (MP Biomedicals, Solon, OH, USA). The DNA was quantified using a Qubit 3™ Fluorometer with the dsDNA HS Assay Kit™ (Thermo Fisher Scientific, Wilmington, DE, USA). DNA was kept at − 20 °C until use.

### Sequencing and Microbial Community Analysis

The DNA was sequenced using an Illumina MiSeq Sequencer at the Institute of Parasitology and Biomedicine López-Neyra (IPBLN-CSIC, Granada, Spain). The prokaryotic (bacteria + archaea) communities were sequenced using the primer pairs Pro341F and Pro805R [34]. The PCR reaction conditions for amplification were as follows: an initial denaturation at 98 °C for 2 min, followed by 35 cycles. The annealing temperature started at 65 °C and decreased by 1 °C each cycle until it reached 55 °C, where it remained for the rest of the cycles, each lasting 15 s. The extension was carried out at 68 °C for 30 s. Library preparation was conducted by the sequencing center. Raw reads were analyzed using QIIME2 v2023.7 following the methods described earlier [35]. In short, sequence reads were dereplicated and merged to generate representative variants known as amplicon sequence variants (ASVs) through the DADA2 method [36]. These ASVs were then matched to the SILVA 138 database within QIIME2 employing the Naïve Bayes classifier [35]. An average of 56,784 high-quality sequences per sample were obtained. Singletons were removed using QIIME2 v2023.7 to reduce noise and improve the reliability of the resulting ASVs.

Analyses of alpha- and beta-diversity indices were done using the “Phyloseq” package in R as described by [35]. Differences in alpha and beta diversity between years (2021 vs. 2022), orientations (south vs. north), and their interaction were tested by analysis of variance (ANOVA) and permutational analysis of variance (PERMANOVA), respectively. For alpha diversity, values of alpha-diversity indices (number of ASVs and values of the Shannon and Simpson indices) were computed for each sample in the dataset. Beta-diversity analysis included a principal coordinates analysis (PCoA) analysis on unweighted UniFrac distances. Additionally, the non-parametric analysis of similarity (ANOSIM) was used to look for differences in beta diversity of the prokaryotic community between years for each lake [37]. Prior to calculating ANOSIM, the three replicate samples from each lake were averaged. This averaging step creates a single composite sample for each lake and year, reducing within-lake variability and focusing on between-year comparisons. ASVs with relative abundance significantly different among years for each lake were identified using the DESeq2 analysis [38]. The functional capabilities of the prokaryotic community were predicted using the PICRUSt2 tool following methods by Douglas et al. [39]. For each lake, significant differences in the relative abundance of functional pathways of the Kyoto Encyclopedia of Genes and Genomes (KEGG) between years were calculated using the Welch’s *t*-test [40] and the Benjamini–Hochberg False Discovery Rate (FDR) multiple-test correction [41] using the R package “sgof.” All KEGG Orthology (KOs) groups belonging to each pathway were used for this analysis.

### Statistical Analyses

Significant differences among years for the physicochemical properties measured in the sediment samples were tested by one-way ANOVA followed by post hoc comparison of the means using Tukey’s HSD (*p* < 0.05). The assumption of normality of residuals was assessed using the Shapiro–Wilk test, while homoscedasticity was evaluated using Bartlett’s test.

The effect of the physicochemical properties (Na^+^, NH_4_^+^, K^+^, Mg^2+^, Ca^2+^, NO_3_^−^, PO_4_^3−^, SO_4_^2−^, TC, TOC, TN, TC/TN, TOC/TN, pH, and EC) as abiotic factors controlling the changes in (i) alpha (values of the Shannon index) and (ii) beta (first axis of the PCoA) diversity of the prokaryotic communities and (iii) the relative abundance of each of the differentially abundant prokaryotic genera were assayed by linear mixed effects model with the “lme4” library of R [42]. The “year,” “orientation,” and their interaction were considered random factors. Multicollinearity among the continuous predictors was checked, and models were run with uncorrelated factors (Na^+^, NH_4_^+^, K^+^, Mg^2+^, Ca^2+^, NO_3_^−^, PO_4_^3−^, SO_4_^2−^, TC/TN, and TOC/TN). A correlation plot matrix based on Pearson correlation coefficients for the selected predictors is provided in Supplementary Fig. S3. The ANOVA analysis identified significant effects (*p*-value), while the Shapiro–Wilk test was used to test the normality of the models’ residuals. Differences among the levels of the random categorical factors were assessed by Tukey’s post hoc tests using the “lsmeans” library of R. All statistical analyses were done in R version 4.3.3 (http://www.rproject.org/).

## Results

### Physicochemical Properties

There were significant increases in the concentrations of Na^+^ (86-fold), NH_4_^+^ (111-fold), Ca^2+^ (30-fold), NO_3_^−^ (eightfold), PO_4_^3−^ (474-fold), and SO_4_^2−^ (18-fold) in sediments from all lakes in 2022 compared to 2021 (Supplementary Table S1). Significantly greater K^+^ (69-fold) and Mg^2+^ (38-fold) concentrations were detected in sediments from CAL, RS1, LVG, LAV, and LTB in 2022 compared to 2021 whereas no significant differences in K^+^ and Mg^2+^ contents were observed in PQ1, PQ2, LVM, and LTA between both years. The increases in the inorganic nutrients were of greater magnitude in lakes located in the south (on average 105-fold) compared to the north (on average 56-fold) orientation within the mountain. There were significant increases in the concentrations of TC (1.2-fold), TOC (1.4-fold), and TN (1.6-fold) in the sediments from PQ2, LVM, LVG, LTA, and LTB lakes in 2022 compared to 2021 whereas no statistical differences in these physicochemical properties were detected in CAL, RS1, PQ2, and LAV lakes. Except for LVG and PQ1, the TC/TN ratio values were significantly lower in sediments from all lakes in 2022 compared to the previous year. Significantly lower TOC/TN values were detected in sediments from CAL, RS1, PQ1, PQ2, LAV, and LTB in 2022 compared to 2021 whereas no statistical differences in the values of this ratio were observed in LVM, LVG, and LTA between years (Supplementary Table S1). Significant increases in the values of pH in sediments of the RS1, LVM, and LTB were detected in 2022 compared to 2021 whereas no significant changes for this parameter were found in the rest of the lakes. EC values were significantly greater in sediments of CAL and RS1 in 2022 compared to 2021, whereas the values of EC decreased in the rest of the lakes from 2021 to 2022.

### Alpha and Beta Diversity of Prokaryotic Communities and Abiotic Controls

**Fig. 1:**
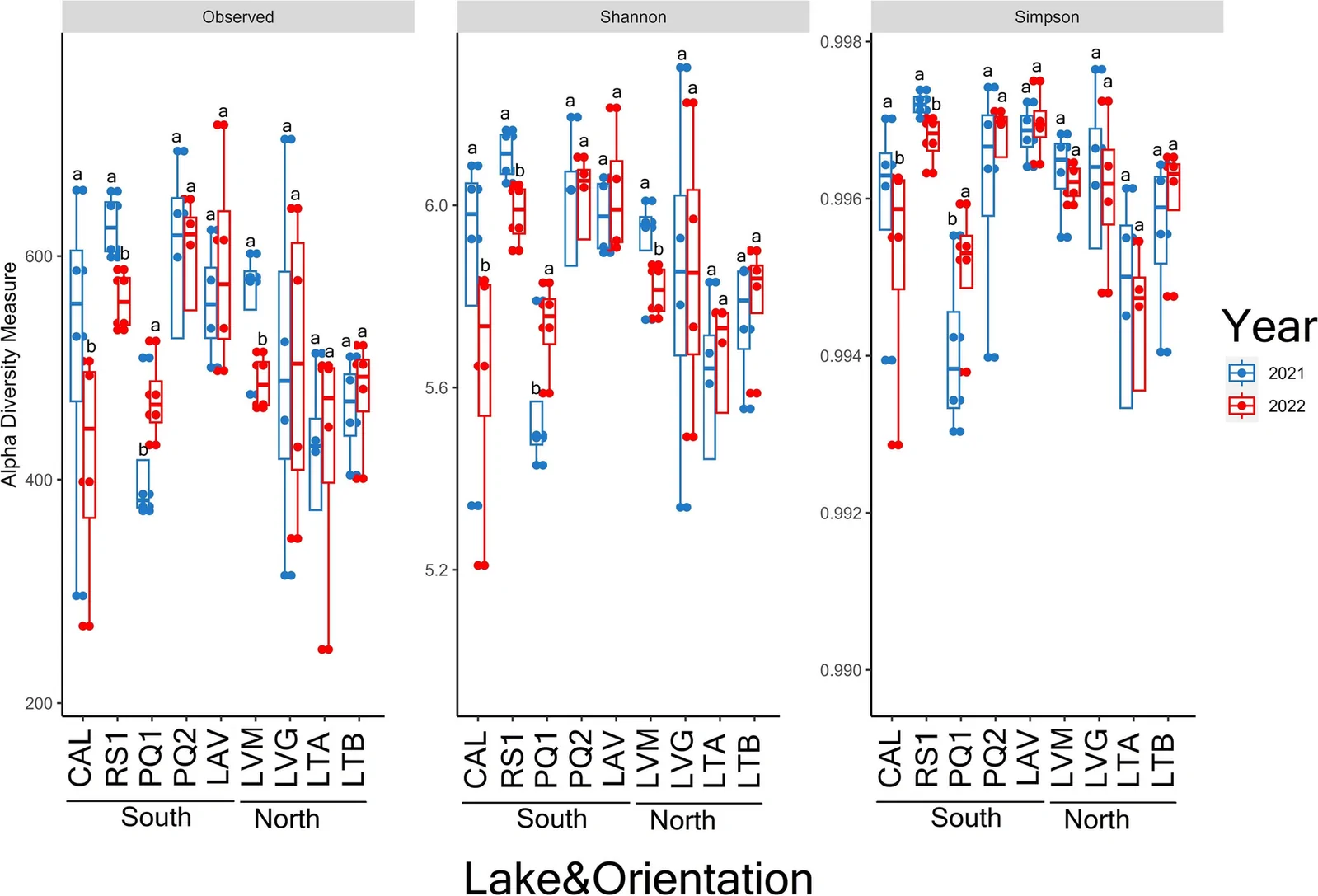
Number of ASVs and values of Shannon and inverse Simpson diversity indices for the prokaryotic community in sediments of the lakes in 2021 and 2022. For each lake, different letters above the bars indicate significant differences between years (Tukey’s HSD, *p* ≤ 0.05). Values are expressed as means with standard error. Lake’s acronyms are defined in Table 1. Differences in alpha diversity between years and orientations were tested by analysis of variance (ANOVA), and *p* values ≤ 0.01 were considered significant

There were significant decreases in the prokaryotic alpha diversity indices between years in three (CAL, RS1, and LVM) out of nine lakes (Fig. 1). Significant increases in the number of ASVs and values of the Shannon and Simpson indices were observed in PQ1 in 2022 compared to 2021. The rarefaction curves showed that all samples reached the saturation phase (Supplementary Fig. S4).

**Fig. 2:**
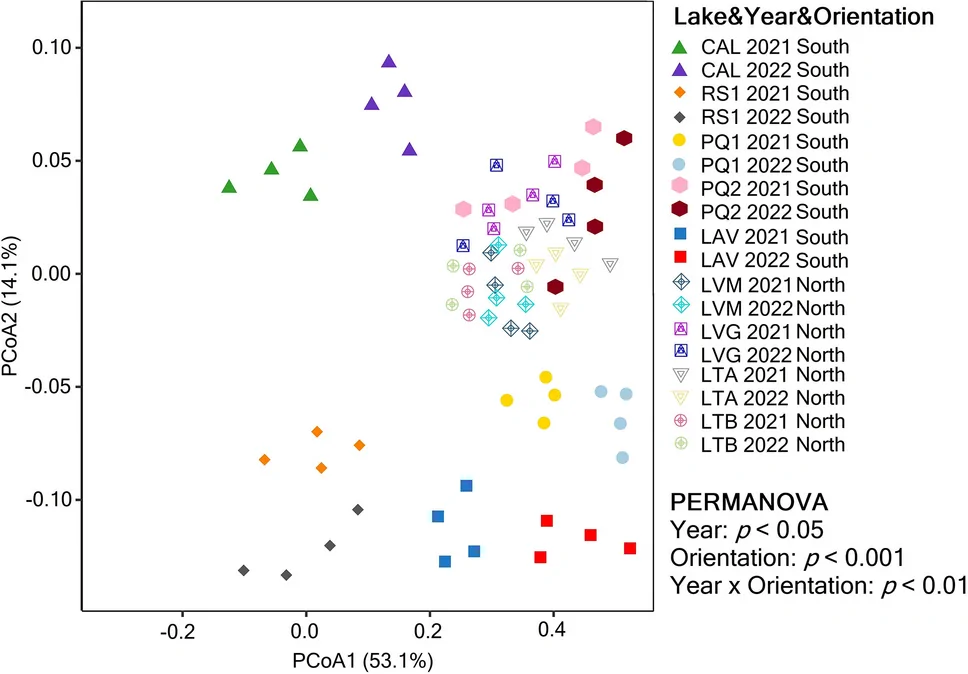
Principal coordinates analysis (PCoA) plots on unweighted UniFrac distances for the prokaryotic community in sediments of the lakes in 2021 and 2022. Differences in community composition between years, orientations, and their interaction were tested by permutational analysis of variance (PERMANOVA), and *p* values ≤ 0.01 were considered significant. Lake’s acronyms are defined in Table 1

PCoA analysis on Bray–Curtis distance together with a PERMANOVA analysis showed significant differences in the composition of prokaryotic communities between year (*p* < 0.05), orientation (*p* < 0.001), and year × orientation interaction (*p* < 0.01) (Fig. 2). A subsequent ANOSIM analysis showed there were no significant differences in beta-diversity between years for the PQ2, LVM, LVG, LTA, and LTB lakes, but that the composition of the prokaryotic community statistically differed between years in the CAL, RS1, PQ1, and LAV lakes (Supplementary Table S2).

Linear mixed effects models revealed the effects of the physicochemical properties as abiotic factors controlling the changes in the alpha and beta diversity of the prokaryotic communities (Table 2). The concentrations of Na^+^, K^+^, Mg^2+^, and Ca^2+^ significantly and positively influenced the alpha- and beta-diversity indices whereas those of NO_3_^−^ and PO_4_^3−^ and values of the TC/TN and TOC/TN ratios had a negative impact on the diversity and composition of prokaryotic communities. The concentration of NO_3_^−^ and PO_4_^3−^ and values of the ratio TC/TN and TOC/TN had a greater negative influence on beta- (*p* < 0.01) than alpha diversity (*p* < 0.05).

**Table 2:** Statistical results of the linear mixed effects models for physicochemical properties as controllers of changes in the alpha and beta diversity of prokaryotic communities in sediments from the lakes. All linear models fulfilled the normal distribution of the residuals (*p* > 0.32, Shapiro’s test). Significant codes: *p < 0.05, **p < 0.01, ***p < 0.001; NS, not significant. The explained variance (*R*^2^) of each predictor was calculated as sums of squares for each variable × 100 / sums of squares for all variables. The coefficient estimate (β) for each predictor is presented. EC, electrical conductivity

	Prokaryotic community					
	Alpha diversity			Beta diversity		
	Coefficient estimates, β	Explained variance, *R*^2^ (%)	Significance levels (*p*-value)	Coefficient estimates, β	Explained variance, *R*^2^ (%)	Significance levels (*p*-value)
Na^+^	0.60	2.45	*	0.51	2.41	*
NH_4_^+^	0.12	0.28	NS	0.26	0.12	NS
K^+^	0.49	3.32	*	0.64	3.95	*
Mg^2+^	0.54	4.34	*	0.61	2.14	*
Ca^2+^	0.63	3.21	*	0.48	3.77	*
NO_3_^−^	− 0.70	6.32	*	− 0.77	9.21	**
PO_4_^3−^	− 0.61	5.65	*	− 0.81	8.86	**
SO_4_^2−^	0.08	0.34	NS	0.12	0.55	NS
pH	0.06	0.12	NS	0.04	0.22	NS
EC	0.11	0.21	NS	0.14	0.52	NS
TC/TN	− 0.55	6.45	*	− 0.75	9.23	**
TOC/TN	− 0.57	6.66	*	− 0.72	10.21	**
Year	-	8.42	*	-	11.23	**
Orientation	-	9.42	*	-	15.16	***
Year × orientation	-	7.31	*	-	5.23	*

### Prokaryotic Community Composition and Differentially Abundant Taxa

On average, Proteobacteria (36.8% ± 10.1) was the dominant phylum across all lakes and years, followed by Bacteriodetes (14.3% ± 5.6), Chloroflexi (12.9% ± 5.1), Actinobacteria (10.7% ± 3.2), and Acidobacteria (9.2% ± 3.4) (Supplementary Fig. S3A). An overview of the main families and genera in the lake’s sediments is shown in Supplementary Fig. S5B and S5C, respectively.

**Fig. 3:**
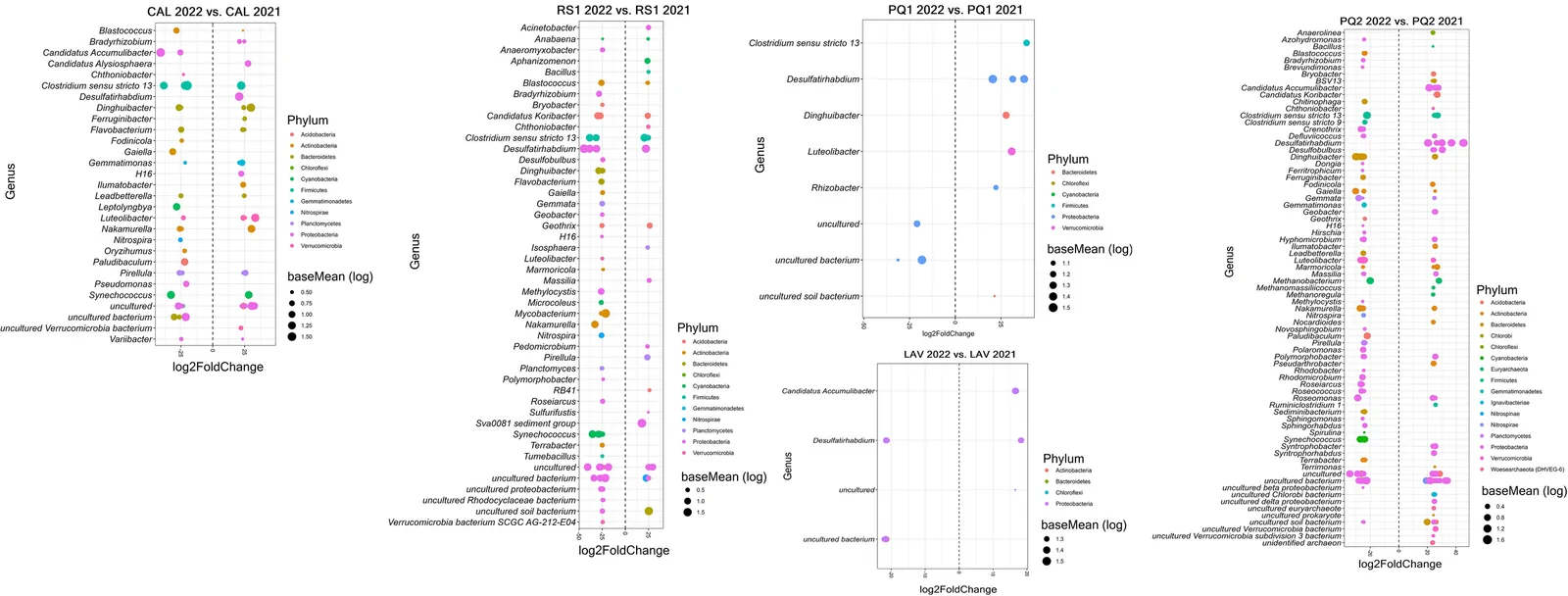
Differential abundance ASVs between years (2022 vs. 2021) for each of the lakes located in the north orientation of Sierra Nevada. Genera showing differentially abundant ASVs are included. The fold change is shown on the X axis, and genera are listed on the Y axis. Each colored dot represents an ASV that was identified by DESeq2 analysis as significantly differentially abundant between years (*p* ≤ 0.05). Lake’s acronyms are defined in Table 1

**Fig. 4:**
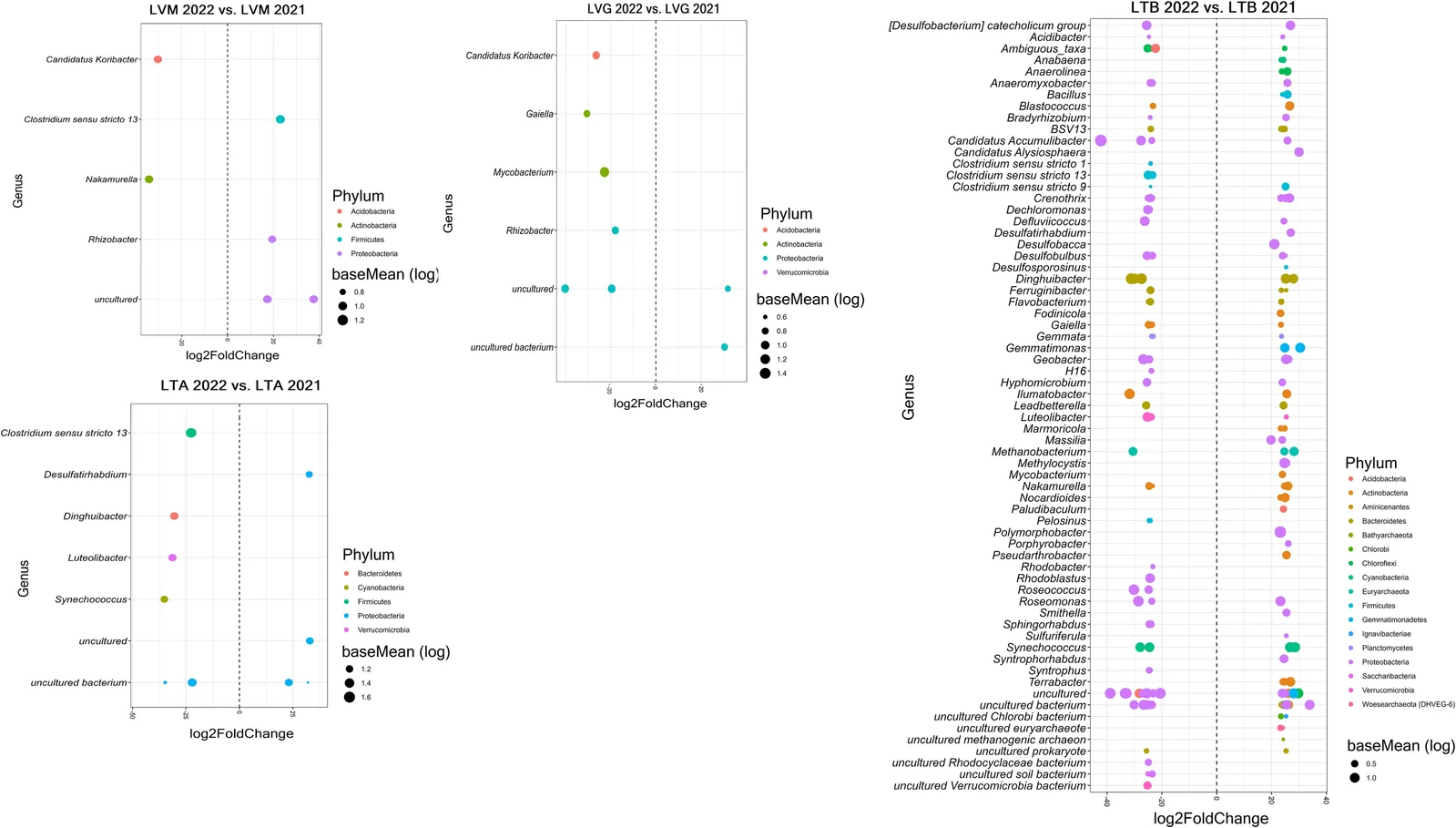
Differential abundance ASVs between years (2022 vs. 2021) for each of the lakes located in the south orientation of Sierra Nevada. Genera showing differentially abundant ASVs are included. The fold change is shown on the X axis, and genera are listed on the Y axis. Each colored dot represents an ASV that was identified by DESeq2 analysis as significantly differentially abundant between years (*p* ≤ 0.05). Lake’s acronyms are defined in Table 1

Differentially abundant ASVs between years (2022 vs. 2021) were identified to provide additional insights into significant variations in the prokaryotic community in sediments of each lake (Figs. 3 and 4). Significantly enriched or depleted ASVs were detected in all lakes but their number and taxonomic affiliation varied depending on the lake. For example, a greater number of responsive genera between years were detected in CAL (29), RS1 (46), PQ2 (75), and LTB (67) compared to PQ1 (8), LAV (4), LTA (7), LVM (5), and LVG (6). Overall, there were a total of 19 prokaryotic genera (e.g., *Acinetobacter*, *Bacillus*, *Ilumatobacter*, *Pedomicrobium*, and *Terrimonas*) containing ASVs that were significantly enriched in 2022 compared to 2021. A total of 25 prokaryotic genera (e.g., *Azohydromonas*, *Chitinophaga*, *Nitrospira*, *Pseudomonas*, and *Tumebacillus*) contained ASVS that were statistically depleted in 2022 compared to the previous year. In general, there were a total of 39 prokaryotic genera (e.g., *Blastococcus, Clostridium*, *Gemmata*, *Geobacter*, and *Rhizobacter*) whose differentially abundant ASVs between years were both enriched and depleted within the same genus, thus suggesting annual effects on these specific genera were species-specific (Fig. 3 and Fig. 4).

### Abiotic Drivers Controlling Changes in the Relative Abundance of Differentially Abundant Taxa

Linear mixed effects models revealed the effects of the physicochemical properties as abiotic factors controlling the changes in the relative abundance of each of the differentially abundant prokaryotic genera between years (2022 vs. 2021) (Supplementary Table S3). Only genera significantly influenced by at least one of the physicochemical properties are shown in Supplementary Table S3. The concentration of Na^+^ negatively influenced the *Desulfobulbus*, *Dongia*, *Hirschia*, *Planctomyces*, and *Sediminibacterium* genera. Other physicochemical properties such as the concentration of NH_4_^+^, NO_3_^−^, SO_4_^2−^, PO_4_^3−^, and Mg^2+^ had both a positive or negative influence on specific and different prokaryotic genera such as *Bacillus*, *Illumatobacter*, *Nocardioides*, *Microcoleus*, *Ozyzihumus*, and *Pedomicrobium*. The relative abundance of *Anaeromyxobacter*, *Dongia*, *Microcoleus*, and *Sphingorhabdus* was negatively affected by the concentration of K^+^. The Ca^2+^ concentration positively influenced the relative abundance of the *Porphyrobacter* genus. The ratios TC/TN and TOC/TN had a positive influence on 18 and 17 genera, respectively, whereas they negatively affected four (*Candidatus* Alysiosphaera, *Ferritrophicum*, *Ferruginibacter*, and *Ilumatobacter*) and seven genera (e.g., *Ferruginibacter*, *Ilumatobacter*, *Methanomassliicoccus*, and *Methanoregula*), respectively.

### Changes in Predicted Prokaryotic Functionality Between Years

**Fig. 5:**
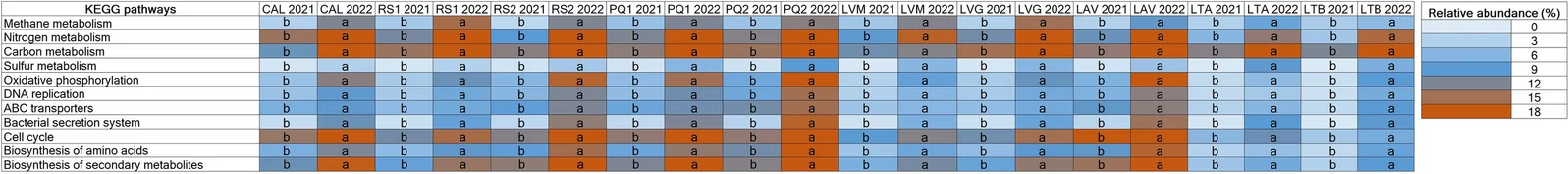
KEGG pathways showing significant differences in their relative abundance (%) between years for each lake. For each lake and KEGG pathway, different letters indicate significant differences between years (Tukey's HSD, *p* ≤ 0.05). Values are expressed as mean with standard error. Lake’s acronyms are defined in Table 1

There were significant increases in the relative abundance of 11 KEGG pathways (methane metabolism, nitrogen metabolism, carbon metabolism, sulfur metabolism, oxidative phosphorylation, DNA replication, ABC transporters, bacterial secretion system, cell cycle, biosynthesis of amino acids, and biosynthesis of secondary metabolites) in all lakes in 2022 compared to 2021 (Fig. 5). These increases were more pronounced in the CAL, RS1, PQ1, PQ2, and LAV lakes compared to the rest of lakes.

## Discussion

This study contributes to filling an information gap on how atmospheric events, such as dust transport, impact on microbiota of remote aquatic ecosystems, considered as excellent sensors of global change. Here, we show that strong intrusions of Saharan dust to high-mountain lakes of Sierra Nevada alter sediment nutrient availability with subsequent impacts on the diversity, composition, and predicted functionality of prokaryotic communities. The extent of changes in nutrient availability and beta diversity appears to be controlled by the orientation of the lakes within the mountain. Greater increases in nutrient concentrations and changes in the composition of prokaryotic communities were observed in lakes located in the south compared to the north orientation of Sierra Nevada, likely due to the former being more affected by the atmospheric deposition episode. We found strong and significant associations between specific physicochemical properties and the relative abundance of prokaryotic genera, thus identifying responsive taxa to atmospheric inputs and key abiotic drivers controlling sediment microbiome variations in these high-mountain lakes. Together, our findings not only provide information on microbiome diversity and its environmental drivers at the local/regional level but also are of general interest as these show that severe atmospheric dust intrusions to remote high-mountain lakes can have significant effects on nutrient availability and prokaryotic communities. This information contributes to understanding how these freshwater ecosystems face future environmental changes such as the increasing atmospheric transport of Saharan dust northwards [2, 43].

### Impacts on Nutrient Availability

The severe Saharan dust intrusion to the Iberian Peninsula that occurred in spring 2022 resulted, as hypothesized, in significant increases in nutrient availability in sediments of nine high-mountain lakes of Sierra Nevada compared to a previous year with lower atmospheric dust deposition (Supplementary Table S1). Although these increases in the concentration of specific nutrients were detected in all lakes in 2022 vs. 2021, they were of greater magnitude in lakes located in the south compared to the north orientation within the mountain. This could be explained considering that the lakes located in the south orientation are expected to receive a greater concentration of nutrients by northward atmospheric dust intrusions compared to lakes located in the north orientation. Although high-mountain lakes in this study were located within a small mountainous area, our results suggest that their specific location in the mountain determines the magnitude of atmospheric dust depositions on sediment nutrient availability, which agrees with previous reports showing the deposition of particles from Saharan dust intrusions is associated with south or southwest winds and therefore greater in the south orientation of Sierra Nevada [2]. It is important to note that high-mountain lakes in this Mediterranean region have been subjected to varying Saharan dust deposition events for decades (Supplementary Fig. S1; [17]), and legacy effects on physicochemical parameters are inherent in the measurements. Despite this annual and continuous exposition to dust intrusions, our 2-year study showed that when these events are of high intensity and large geographical extent, they can greatly alter nutrient availability. Atmospheric Saharan dust depositions to high-mountain lakes of Sierra Nevada have been previously reported to be a significant source of inorganic (mainly PO_4_^3−^) and organic nutrients [2], but our results extend those findings by showing that they can introduce high concentrations of different inorganic macro- and micro-nutrients including Na^+^, K^+^, Mg^2+^, NH_4_^+^, Ca^2+^, NO_3_^ͨ−^, PO_4_^3−^, SO_4_^2−^, TC, and TOC. The observed decreases in EC in lake sediments from 2021 to 2022, despite a substantial increase in Na^+^ concentration, may be attributed to complex interactions among various ions, changes in sediment composition, and/or a potential dilution effect caused by the significant rise in diverse ion concentrations during this period. These factors collectively influence conductivity values and highlight the multifaceted nature of ion dynamics in aquatic ecosystems. Our results also reveal that these strong atmospheric episodes greatly contribute to increases in the availability of TN in sediments of the lakes, as shown by significant decreases in the values of the TC/TN ratio in 2022 compared to the previous year (Supplementary Table S1). This is in line with previous studies showing that reactive N species (Nr), mainly in the form of NO_3_^−^, are introduced to high-mountain lakes of Sierra Nevada via atmospheric depositions [44]. These alterations of the natural N cycle in high-mountain freshwater ecosystems are of great environmental concern due to their potentially detrimental impact on ecosystem functioning, resilience, and biodiversity [11, 20]. A previous study demonstrated that La Caldera Lake, a high-mountain lake of Sierra Nevada that was included in this study, acts as a “dissimilative biological N-pump” responsible for N losses via gas emissions in sediments of this ecosystem [20]. Yet little is known about the temporal dynamics and cycling of inorganic and organic nutrients following strong atmospheric dust deposition events in Mediterranean high-mountain lakes which deserve further attention and can help better understand the biogeochemical consequences of these Saharan dust episodes.

### Impacts on Microbial Diversity and Composition

We found that inputs of specific nutrients through strong Saharan dust deposition control changes in the diversity (Fig. 1) and composition (Fig. 2) of prokaryotic communities in sediments of high-mountain lakes of Sierra Nevada. The changes in beta diversity were mainly observed in lakes located in the south orientation of the mountain which were greatly affected by the atmospheric dust deposition event. In general, and agreeing with our hypothesis, the impacts on alpha diversity appear to be lake-specific as decreases (3 out of 9 lakes), increases (1 out of 9 lakes), or no changes (5 out of 9 lakes) in alpha diversity of prokaryotic communities were observed between years. The significant contribution of different physicochemical properties to variations in the alpha and beta diversity of prokaryotic communities highlights the ability of the sediment microbiome of high-mountain lakes of Sierra Nevada to respond to external inputs of different nutrients via Saharan dust intrusion events (Table 2). This is in line with results in previous studies showing that Saharan dust can impact the abundance of bacterial communities using ex situ microcosm experiments in reservoirs [14] and in situ experiments in a high-mountain lake of Sierra Nevada (La Caldera Lake; Vila et al. submitted). The negative influence of NO_3_^−^ and PO_4_^3−^ concentrations and values of the TC/TN and TOC/TN ratios on alpha diversity agree with previous studies that reported decreases in the diversity of prokaryotic communities in freshwater and marine ecosystems affected by these inorganic and organic nutrients [20, 45]. Our findings extend those from previous studies reporting that Saharan dust intrusion events can impact the composition of the bacterioneuston in high-mountain lakes of the Pyrenees [22–24] and Austrian and Italian Alps [25, 26]. Variations in the composition of the denitrifying communities have been previously observed in a high-mountain lake of Sierra Nevada which was linked to variations in Nr via wet depositions [20].

### Impacts on Unique Prokaryotic Species

The alteration of the sediment prokaryotic microbiome was further supported by detecting specific prokaryotic taxa whose relative abundance significantly varied between years (Figs. 3 and 4), as hypothesized, which was controlled by specific physicochemical properties. Although the taxonomic affiliation of the genera containing differentially abundant ASVs varied between lakes, altogether, we identified a total of 19 and 25 genera whose relative abundances were significantly enriched or depleted in the lakes, respectively. Enriched genera such as *Acinetobacter*, *Bacillus*, *Ilumatobacter*, *Pedomicrobium*, and *Terrimonas* were positively influenced by the concentrations of NH_4_^+^, NO_3_^−^, and PO_4_^3−^ and values of the ratios TC/TN and TOC/TN. However, these inorganic and organic nutrients had also a negative influence on depleted genera such as *Azohydromonas*, *Chitinophaga*, *Nitrospira*, *Pseudomonas*, and *Tumebacillus*. Previous studies have reported that species belonging to *Bacillus* and *Pseudomonas* can solubilize PO_4_^3−^ [46] whereas *Nitrospira* is known to contribute to the nitrification process in natural environments [47]. *Acinetobacter* and *Ilumatobacter* species have also ecological importance as they can contribute to denitrification [48] and degradation of organic compounds [49] in lake sediments. Therefore, all these changes in the relative abundance of specific genera following dust deposition are expected to impact nutrient cycling in sediments of high-mountain lakes of Sierra Nevada. Of note, we identified a total of 109 significant interactions between specific nutrients and prokaryotic genera, thus highlighting the strong impact of these severe atmospheric episodes on prokaryotic communities in these freshwater ecosystems. We also found that the response of 39 prokaryotic genera was species-specific as they had both enriched and depleted ASVs within the same genera. These results highlight that the microbiome impacts of dust deposition on prokaryotic communities can be both widespread across the prokaryotic microbiome and species-specific. It cannot be ruled out that factors other than the input of nutrients via dust deposition may have contributed to variations in the taxonomic composition of microorganisms between years in the lakes. However, given (1) the strong impact of the strong Saharan deposition on nutrient availability in sediments of the lakes (supported by measuring changes in numerous physicochemical properties between years in this study), (2) the results of the linear mixed-effects models showing that abiotic factors controlled variations in the relative abundance of differentially abundant taxa, and (3) the remote location of the high-mountain lakes, altogether, led us to assume that factors, other than the Saharan dust deposition event, had minor impacts (if any) on the microbial communities in the high-mountain lakes.

### Impacts on Predicted Functionality

The severe Saharan dust intrusion to high-mountain lakes of Sierra Nevada resulted in increases in predicted microbial functionality which were more pronounced in lakes located in the south orientation compared to those in the north orientation (Fig. 5), as hypothesized. The significantly enriched prokaryotic functions were assigned to different biogeochemical cycles (e.g., carbon, nitrogen, sulfur, and methane cycles) and general metabolic pathways (e.g., biosynthesis of amino acids and secondary metabolites). Increased nutrient availability in sediments of the lakes following the dust deposition event can be used by microorganisms in different processes and metabolic activities, thus altering the functionality of the sediment microbiome. This is important as it shows that the sediment prokaryotic microbiome can adapt rapidly to high inputs of nutrients and likely contribute to its cycling. In particular, enhanced carbon and nitrogen metabolism suggest increased primary productivity and nutrient cycling, while the increases in oxidative phosphorylation indicate heightened cellular respiration and energy production. The upregulation of biosynthesis of amino acids and secondary metabolite in 2022 compared to 2021 suggests that microbial communities were enhancing their growth and adapting their metabolic activities. These increases in microbial activity often lead to changes in community composition, as observed in this study. We acknowledge that predictions of microbial functionality using tools such as PICRUSt2 should be taken with care [50] as other methods based on cDNA or RNA can provide more accurate information about microbial community functions and metabolic activity. However, the tool remains useful for predicting general microbial functions of environmental studies [50].

### Environmental Implications

**Fig. 6:**
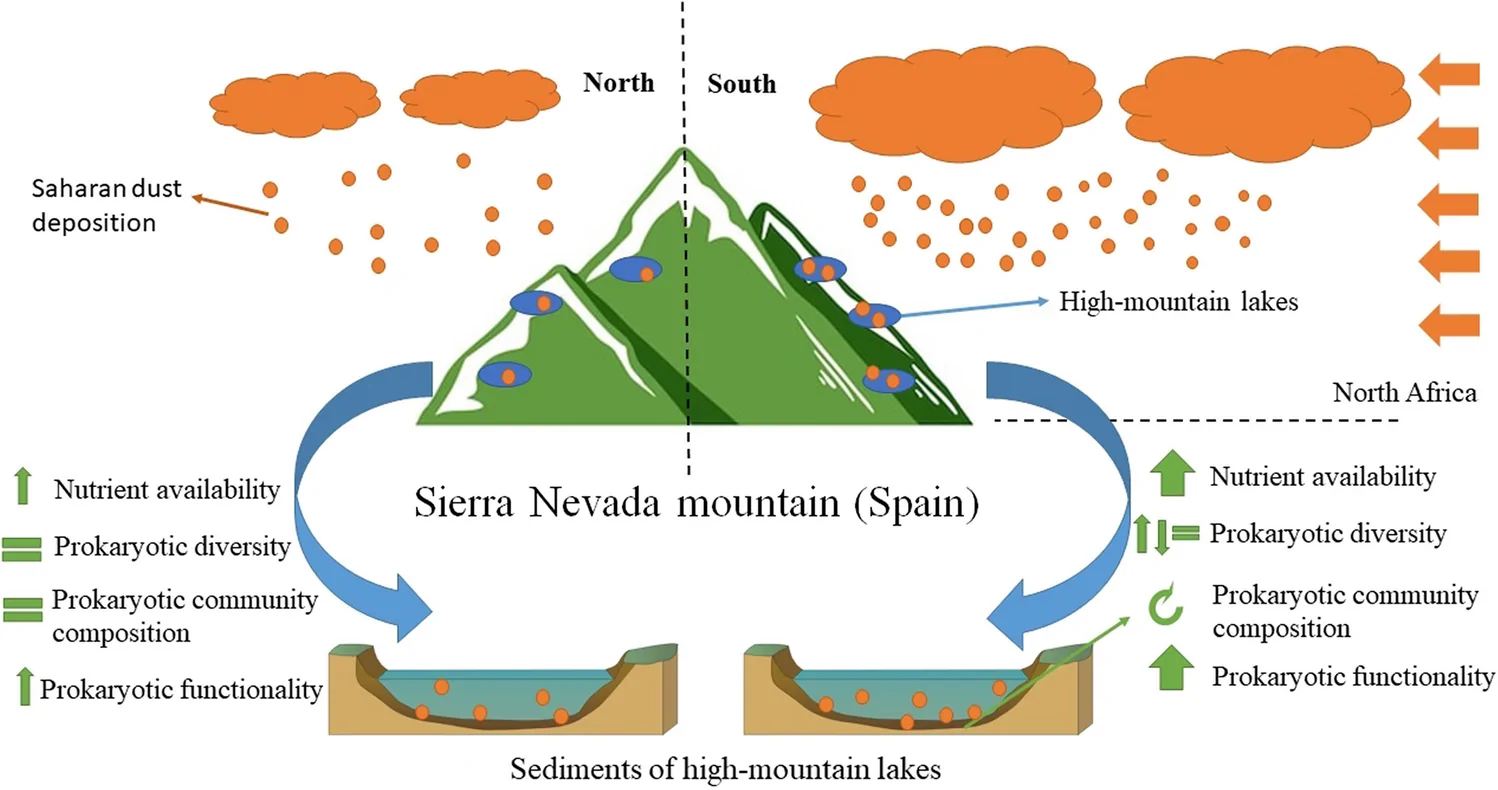
Conceptual model showing the impacts induced by strong Saharan dust deposition events on physicochemical properties, microbial diversity, and functionality in sediments of high-mountain lakes of Sierra Nevada (Spain)

Our results, summarized in Fig. 6, extend previous findings which reported that dust inputs can alter biogeochemical functioning (e.g., carbon and nitrogen cycling) in the water of alpine and polar lakes [2]. The increases in the relative abundance of the microbial nitrogen metabolism pathway agree with previous studies that reported increases in nitrification and denitrification processes in high-mountain lakes and rivers of Sierra Nevada affected by atmospheric depositions of Nr [20, 21].

Strong Saharan dust deposition events in Mediterranean high-mountain lakes have become more frequent over the last decades with unknown ecological consequences. Our study contributes to elucidating how microbes respond to these environmental fluctuations and to what extent severe atmospheric dust deposition episodes can induce variations in the composition and diversity of microbial communities and their contributions to different biogeochemical cycles. This study shows that strong Saharan dust deposition events in high-mountain lakes of a model Mediterranean mountain area (Sierra Nevada, Spain) increase nutrient availability with subsequent impacts on the diversity, composition, and predicted functionality of sediment prokaryotic communities. We found significant strong associations between specific nutrients and changes in the composition of prokaryotic communities. Responsive prokaryotic taxa to a severe Saharan dust deposition event were identified, and their relative abundances were linked to specific physicochemical properties. Our results show that the extent of changes in nutrient availability and beta diversity of prokaryotic communities is influenced by the orientation of the lakes on the mountain slopes, with greater changes in lakes located in the south compared to the north orientation likely because the former was more affected by the atmospheric northward dust intrusion episode. Together, our findings, summarized in Fig. 6, suggest that severe dust intrusions to Mediterranean high-mountain lakes located in southern Europe can have significant biogeochemical and biodiversity consequences through changes in nutrient availability and prokaryotic communities.

## Supplementary Information

Below is the link to the electronic supplementary material.

Supplementary file1 (JPG 5574 KB)

Supplementary file2 (PDF 2720 KB)

Supplementary file3 (XLSX 22 KB)

## Data Availability

Raw sequence data were deposited into the National Library of Medicine (NCBI) under BioProject PRJNA1103194.
