# Supplementary material for: Strong Saharan Dust Deposition Events Alter Microbial Diversity and Composition in Sediments of High-Mountain Lakes of Sierra Nevada (Spain)
Source: Microb Ecol. 2024 Jul 27;87(1):99. doi: 10.1007/s00248-024-02416-w (PMC11283396; doi:10.1007/s00248-024-02416-w)
Supplement: Supplementary file 2 — Supplementary file2 (PDF 2720 KB) [file 248_2024_2416_MOESM2_ESM.pdf]

Supplementary Material

Strong Saharan dust deposition events alter microbial diversity and composition in sediments of high-mountain lakes of Sierra Nevada (Spain)

Authors:

Antonio Castellano-Hinojosa[1,2*], Germán Tortosa[3], Alejandra Fernández-Zambrano[1], David Correa-Galeote[1,2], Eulogio J. Bedmar[3], Juan M. Medina-Sánchez[1,4*]

Affiliations:

[1]Instituto Universitario de Investigación del Agua, Universidad de Granada, Granada, Spain

[2]Departamento de Microbiología, Facultad de Farmacia, Universidad de Granada, Granada, Spain

[3]Departamento de Microbiología del Suelo y Sistemas Simbióticos, Estación Experimental del Zaidín, Consejo Superior de Investigaciones Científicas, Granada, Spain

[4]Departamento de Ecología, Facultad de Ciencias, Universidad de Granada, Granada, Spain

*Corresponding authors:

A. Castellano-Hinojosa

email: ach@ugr.es

J.M. Medina-Sánchez

email: jmmedina@ugr.es

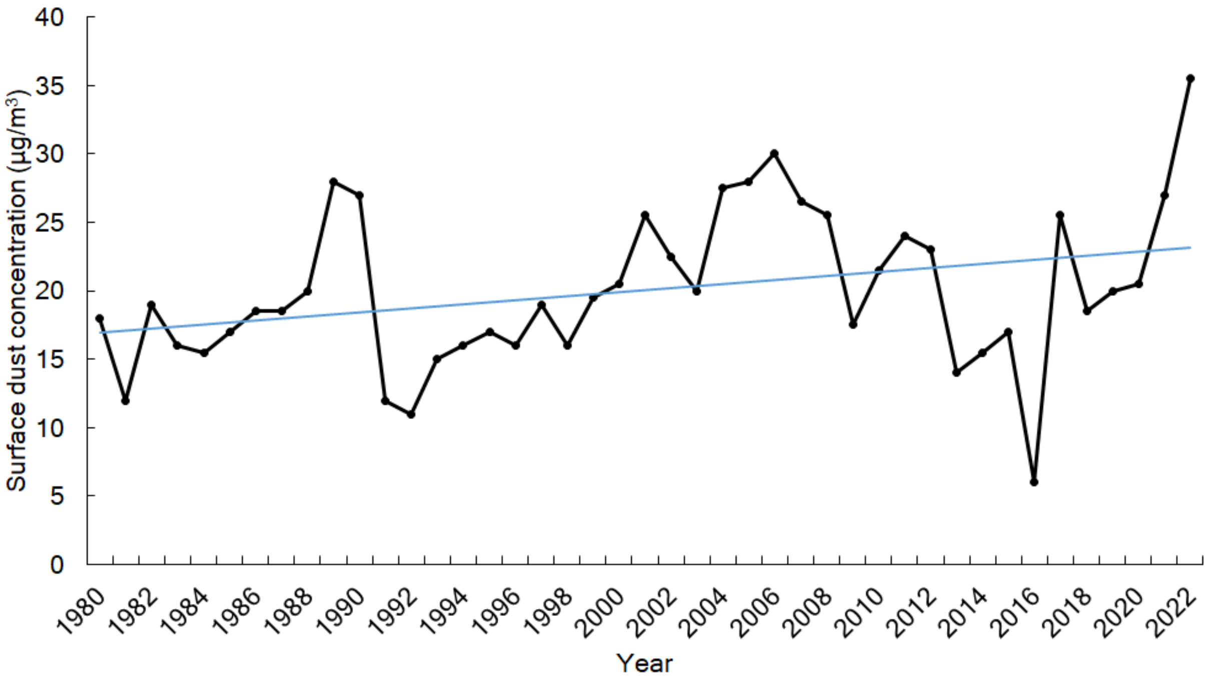

Supplementary Fig. S1. Interannual variability of surface dust concentration between 1980 and 2022. The linear trend is shown in blue. Data obtained from the MERRA-2 pixel containing Granada and part of Sierra Nevada (SmartEcomountains 2022).

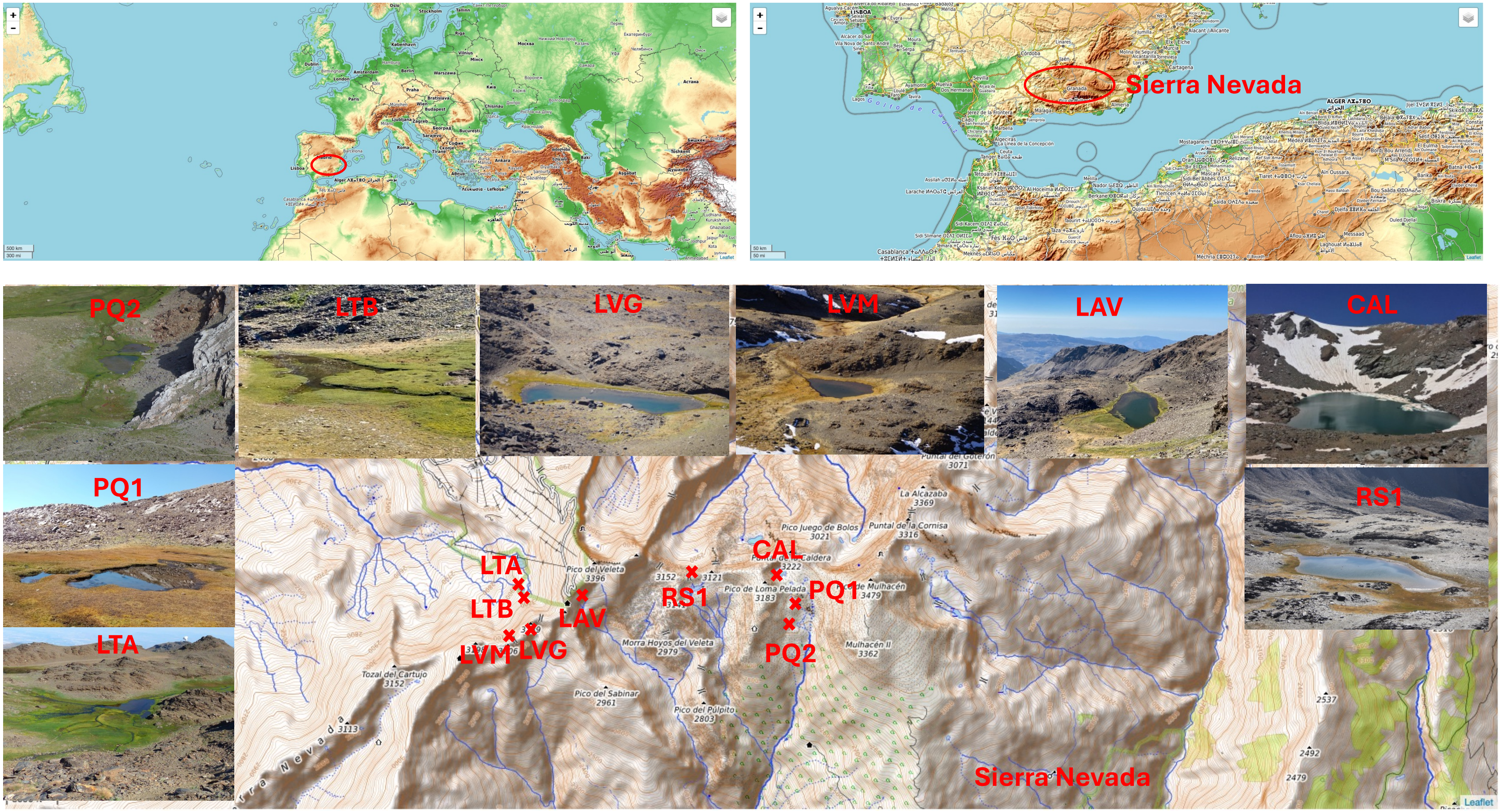

Supplementary Fig. S2. Map of the location of the sampling lakes included in this study. The map was created using Leaflet and images of the lakes were taken from https://lagunasdesierranevada.es. We acknowledge Eulogio Corral Arredondo (PQ2), Rafael M. Cuesta (RS1, LVM, and LVG), Juan Manuel Tapia (LTA), Trinidad Luque Martín (LTB), Aitor Garcia de Frutos (LAV), Alicia Carrasco Navas (CAL), and Alejandra Fernández-Zambrano (PQ1) for the pictures of the high-mountain lakes. Lake´s acronyms are defined in Table 1.

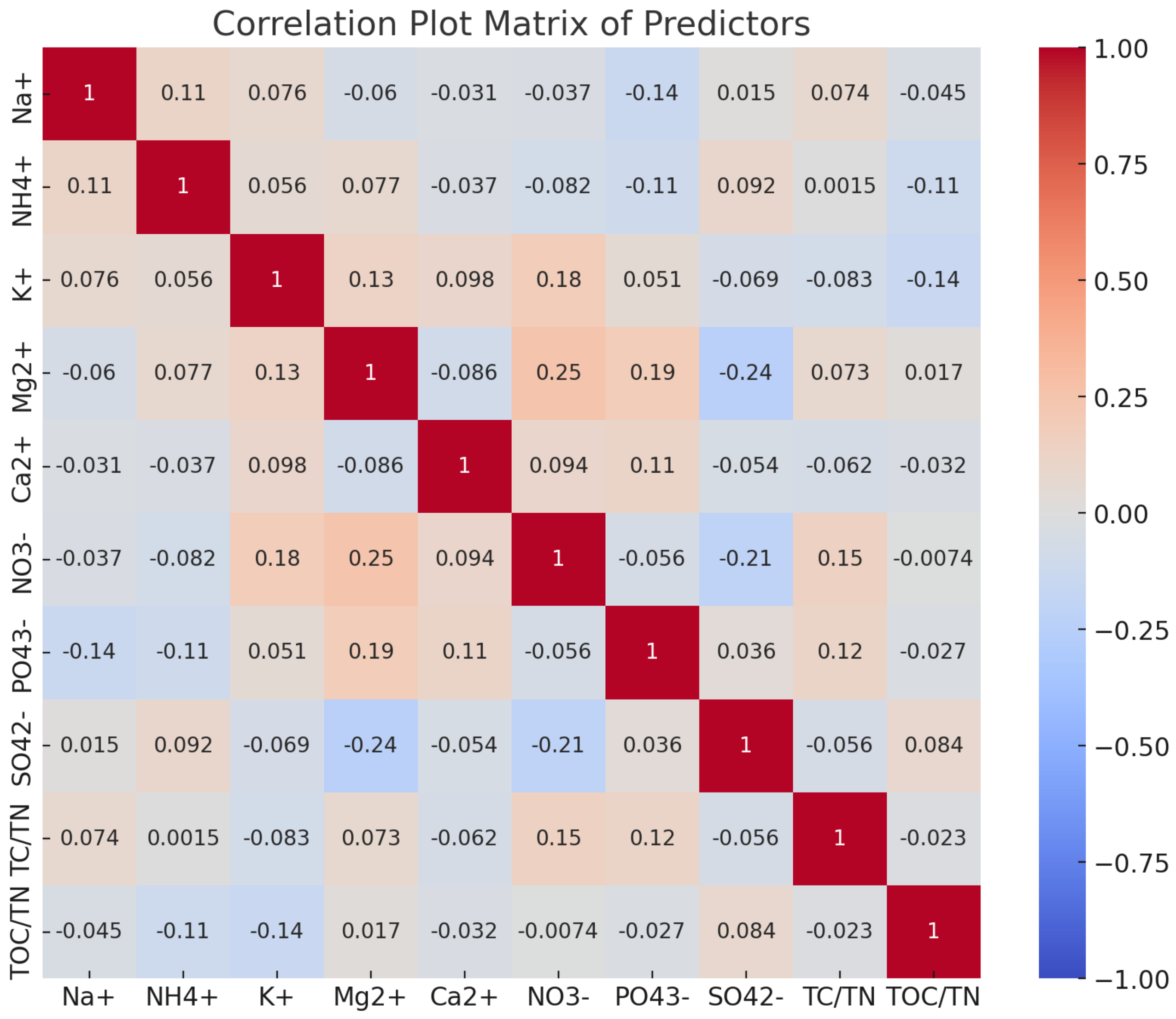

Supplementary Fig. S3. Correlation plot matrix for the selected predictors used in the linear mixed effects models: Na^+, NH_4^+, K^+, Mg^{2+}, Ca^{2+}, NO_3^-, PO_4^{3-}, SO_4^{2-}, TC/TN, and TOC/TN. Each cell in the matrix represents the Pearson correlation coefficient between a pair of predictors, with values ranging from -1 to 1. Positive values (closer to 1) indicate a strong positive correlation, whereas negative values (closer to -1) indicate a strong negative correlation. Values near 0 suggest weak or no correlation between the predictors. The color gradient from blue to red helps visualize the strength and direction of the correlations, where blue indicates negative correlations, red indicates positive correlations, and the intensity of the color represents the magnitude of the correlation.

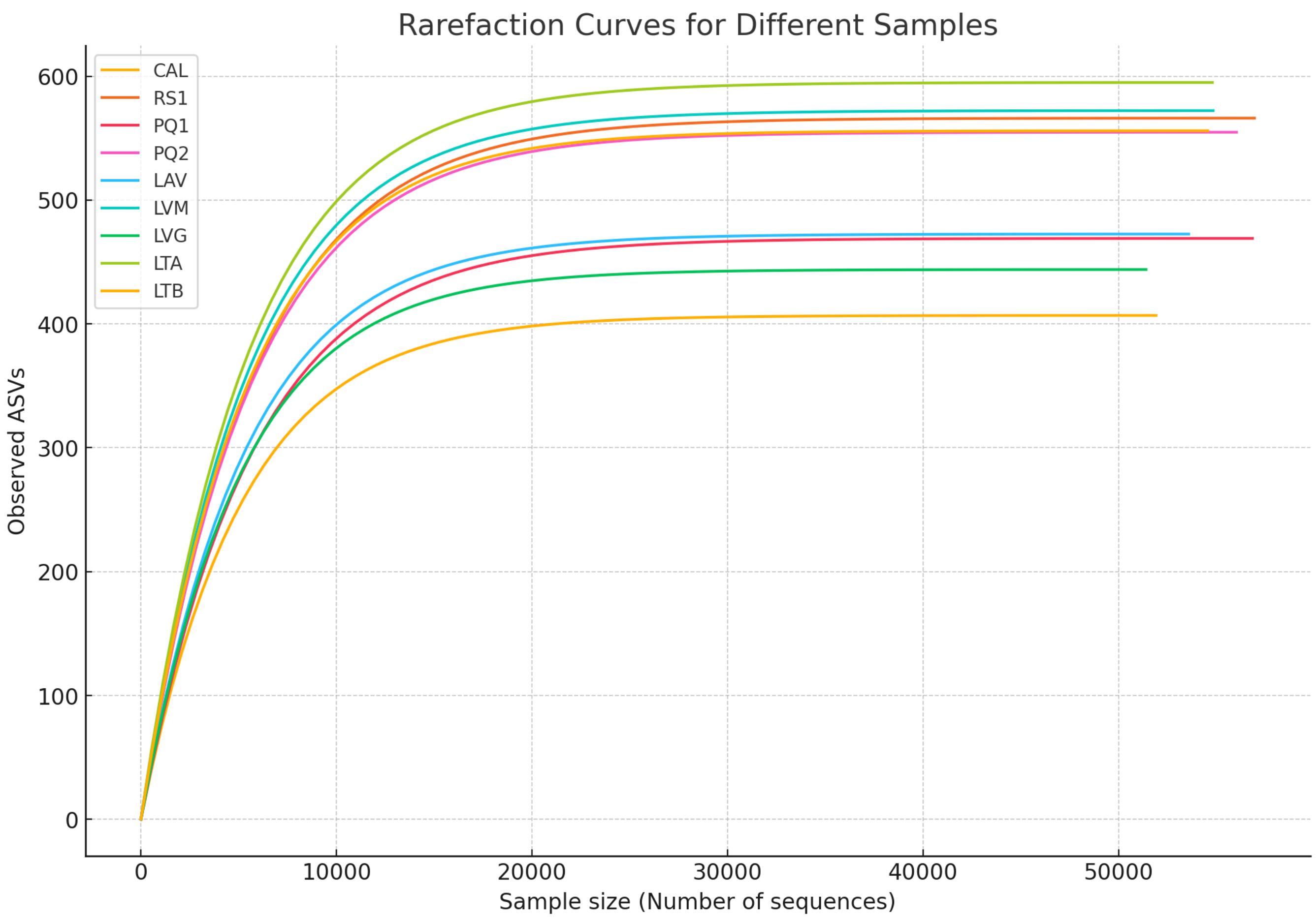

Supplementary Fig. S4. Rarefaction curves for the samples included in this study. Replicates were averaged per sample (n = 4). Lake´s acronyms are defined in Table 1.

Supplementary Table S1. Physicochemical properties in sediments of the lakes in 2021 and 2022. For each lake, values followed by the same lowercase letter are not statistically different among years according to one-way ANOVA followed by post-hoc comparison of the means using Tukey's HSD (p < 0.05). Lake´s acronyms are defined in Table 1. Values are expressed as mean with standard error (n = 4). *n.d.: not detected.

		mg/kg dry sediment										g/kg dry sediment				
**Lake**	**Year**	**Na^+**	**NH_4^+**	**K+**	**Mg^{2+}**	**Ca^{2+}**	**NO_3^-**	**PO_4^{3-}**	**SO_4^{2-}**	**pH**	**EC (µS/cm)**	**TC**	**TOC**	**TN**	**TC/TN**	**TOC/TN**
CAL	2021	2.4±0.4b	0.2±0.2b	3.0±0.7b	2.1±1.0b	15.2±4.9b	2.2±1.1b	n.d.	2.9±0.3b	7.5±0.1a	44±3.6b	5.0±0.2a	3.6±0.1a	0.3±0.1a	18.5±0.7a	13.1±0.7a
CAL	2022	414±108a	66±36a	152±40a	56±22a	760±344a	51.7±15.9a	42.2±17.1a	142±21a	7.8±0.0a	64±2.0a	4.4±0.9a	2.7±0.6a	0.6±0.1a	7.7±0.6b	4.8±0.4b
RS1	2021	2.1±0.1b	11.0±2.7b	3.0±0.8b	1.8±0.5b	6.9±2.4b	1.7±0.5b	n.d.	6.9±0.7b	7.2±0.1b	54±1.5b	7.8±0.2a	4.0±0.1a	0.5±0.1a	16.0±1.9a	8.3±1.0a
RS1	2022	658±65a	395±75a	466±120a	190±23a	568±73a	25.7±7.6a	85.5±41.1a	499±97a	8.1±0.1a	97±3.0a	8.9±1.1a	3.7±0.4a	0.8±0.1a	11.0±0.8b	4.5±0.4b
PQ1	2021	26.2±8.7b	42.6±7.0b	54.3±38.5a	10.1±6.9a	96.1±64.1b	3.6±1.0b	n.d.	15.3±5.7b	7.0±0.0a	182±17a	215±2.7a	176±1.9a	17.7±0.1a	12.1±0.1a	9.9±0.1b
PQ1	2022	212±50a	368±117a	61.0±30.3a	45.1±19.1a	294±87a	22.3±5.8a	n.d.	87.1±6.9a	6.9±0.1a	23±1.0b	206±10.7a	175±7.2a	14.1±1.5a	14.9±1.1b	12.8±1.2a
PQ2	2021	18.3±2.6b	57.0±27.3b	88.5±42.9a	32.2±15.9a	245±144b	10.7±1.2b	n.d.	40.6±8.0b	7.1±0.0a	219±0.6a	86.0±8.3a	64±2.6a	6.4±0.1a	13.4±0.3a	10.1±0.9a
PQ2	2022	350±7.1a	308±143a	37.5±9.0a	67.4±34.8a	1002±505a	31.9±6.9a	n.d.	182±18a	7.0±0.0a	32±4.9b	60.5±0.5b	43±1.3b	5.9±0.1b	10.3±0.2b	7.3±0.3b
LVM	2021	7.7±3.0b	123±76b	46.8±30.5a	23.8±15.1a	54.0±32.2b	2.8±1.4b	n.d.	139±68b	6.6±0.0b	193±2.9a	40.6±1.1b	25.6±3.3b	3.6±0.1b	11.4±0.4a	7.1±0.8a
LVM	2022	317±42a	360±228a	121±66a	48.7±12.2a	562±117a	27.9±16.4a	39.9±7.3a	1029±278a	7.4±0.1a	88±4.8b	50.4±2.5a	39.4±3.7a	5.5±0.3a	9.2±0.5b	7.3±0.8a
LVG	2021	6.2±0.8b	111±39b	42.2±12.4b	31.6±8.9b	66.3±22.0b	5.2±0.7b	n.d.	25±8.6b	6.9±0.1a	273±8.7a	27.3±0.8a	17.4±0.5a	2.6±0.1a	10.6±0.3a	6.8±0.4a
LVG	2022	398±66a	1942±970a	781±421a	440±266a	1037±550a	15.8±7.2a	40.4±14.2a	2014±1487a	7.4±0.1a	121±7.2b	15.8±3.5b	8.8±3.7b	1.3±0.2b	11.5±1.1a	6.1±1.7a
LAV	2021	3.0±0.9b	1.3±1.0b	2.6±1.2b	3.5±0.8b	10.6±3.3b	3.2±1.6b	n.d.	35.9±20.3b	7.4±0.0a	38±1.2a	16.2±0.5a	11.3±1.5a	1.5±0.0a	11.1±0.1a	6.9±0.1a
LAV	2022	297±7a	732±417a	275±128a	64.4±17.8a	711±87a	13.3±1.2a	172±62a	259±103a	7.6±0.1a	15±1.2b	13.7±1.8a	9.5±1.8a	1.7±0.2a	8.3±0.7b	5.6±0.3b
LTA	2021	13.7±1.1b	135±73b	101±22.2a	70.5±20.6a	162±55b	16.4±1.1b	2.7±2.7b	52.7±27.9b	7.0±0.1a	173±5.3a	30.4±1.3b	22.6±1.3b	2.4±0.1b	12.5±0.1a	9.3±0.1a
LTA	2022	224±53a	338±96a	48.6±21.5a	37.1±12.5a	509±305a	37.7±13.1a	72.3±50.4a	2146±1254a	7.4±0.0a	103±9.2b	70.4±5.5a	62.9±5.9a	7.1±0.4a	10.0±0.2b	8.9±0.6a
LTB	2021	10.3±3.2b	67±24b	52.1±18.2b	20.3±7.1b	47.6±16.5b	5.7±3.0b	n.d.	86.1±34.9b	6.3±0.1b	185±5.4a	54.0±2.1b	39.1±2.3b	4.0±0.1b	13.5±0.1a	9.9±0.9a
LTB	2022	432±67a	2306±328a	761±183a	550±69a	1452±96a	34.0±19.7a	45.3±30.0a	879±65a	7.0±0.1a	62±6.3b	74.8±2.0a	57.9±1.7a	7.6±0.3a	9.9±0.2b	7.7±0.2b

Supplementary Table S2. Significance and similarity based on changes in the relative abundance of ASVs between 2021 and 2022 for each lake using the non-parametric multivariate (ANOSIM) statistical method. Lake´s acronyms are defined in Table 1. Numbers in bold indicate significant effect at $p \leq 0.05$. R values close to 1 indicate dissimilarity between treatments.

Comparison	Prokaryotic community	
	ANOSIM	
	R	*p*
CAL 2021 vs. CAL 2022	0.846	0.003
RS1 2021 vs. RS1 2022	0.786	0.015
PQ1 2021 vs. PQ1 2022	0.689	0.019
PQ2 2021 vs. PQ2 2022	0.212	0.069
LVM 2021 vs. LVM 2022	0.189	0.072
LVG 2021 vs. LVG 2022	0.265	0.089
LAV 2021 vs. LAV 2022	0.940	0.005
LTA 2021 vs. LTA 2022	0.345	0.084
LTB 2021 vs. LTB 2022	0.219	0.077

Supplementary Fig. S5. Relative abundance of prokaryotic ASVs at the phylum (A), family (B), and genus taxonomic levels in 2021 and 2022 in each of the lakes. Phyla, families, and genera with relative abundances greater than 1%, 1%, and 0.7% are shown, respectively. Lake´s acronyms are defined in Table 1.

Supplementary Table S3. Statistical results of the linear mixed effects models for physicochemical properties as controllers of changes in the relative abundance of the differentially abundant prokaryotic genera (Figs. 3 and 4) in sediments from the lakes. All linear models fulfilled the normal distribution of the residuals ($p > 0.39$, Shapiro's test). Significant codes: *p <0.05, **p <0.01, ***p <0.001; NS, not significant. The explained variance (R^2) of each predictor was calculated as sums of squares for each variable × 100 / sums of squares for all variables. Coefficient estimate (β) for each predictor is presented.

References

Smart Ecomountains. 2022. https://smartecomountains.lifewatch.dev/en/aumento-concentracion-polvo-sahariano-sierra-nevada/.
